# Buprenorphine Transdermal Delivery System: Bioequivalence Assessment and Adhesion Performance of Two Patch Formulations

**DOI:** 10.3390/pharmaceutics16101249

**Published:** 2024-09-26

**Authors:** Marcelo Gomes Davanço, Miguel Fortuny, Alejandro Scasso, Jessica Meulman, Fernando Costa, Thalita Martins da Silva, Débora Renz Barreto Vianna, Leonardo de Souza Teixeira, Karini Bruno Bellorio, Ana Carolina Costa Sampaio, Celso Francisco Pimentel Vespasiano

**Affiliations:** 1 Clinical Research Unit, Adium S.A., São Paulo 04794-000, Brazil; marcelo.davanco@adium.com.br (M.G.D.); jessica.meulman@adium.com.br (J.M.); thalita.silva@adium.com.br (T.M.d.S.); debora.vianna@adium.com.br (D.R.B.V.); 2 R&D Department, Amarin Technologies S.A., Buenos Aires C1416BQG, Argentina; miguel.fortuny@amarintech.com.ar (M.F.); alejandro.scasso@amarintech.com.ar (A.S.); 3 Corporate Department of Clinical Studies, Adium S.A. Headquarters, Buenos Aires B1603APO, Argentina; fcosta@raffo.com.ar; 4 Bioequivalence Unit, Instituto de Ciências Farmacêuticas de Estudos e Pesquisas Ltda., Goiânia 74935-530, Brazil; leonardo.teixeira@icf.com.br (L.d.S.T.); karini.bellorio@icf.com.br (K.B.B.); ana.cacula@icf.com.br (A.C.C.S.)

**Keywords:** buprenorphine, transdermal delivery system, patch, opioid, bioequivalence, adhesion, pharmacokinetics

## Abstract

**Background and Objective:** Buprenorphine is an opioid drug indicated for the management of severe and persistent pain. The buprenorphine transdermal patch provides a non-invasive method of rate-controlled drug release, ensuring constant and predictable drug plasma levels over an extended period. This study aimed to assess the bioequivalence, skin adhesion non-inferiority, and tolerability of two buprenorphine transdermal patches to meet the regulatory requirements for the registration of a generic product in Brazil. **Methods:** A randomized, single-dose, two-period, two-sequence crossover trial was performed involving healthy subjects of both genders. The subjects received a single dose of either the test formulation or the reference formulation (Restiva^®^), separated by a 29-day washout period. For pharmacokinetic analysis, blood samples were collected up to 12 days post-dose and quantified using a validated bioanalytical method. Skin adhesion was assessed over a 7-day period (dosing interval) following patch application. Seventy-six subjects were enrolled and fifty-two completed the study. **Results and Conclusion:** The 90% confidence intervals for Cmax, AUC_0–t_, and partial AUCs were within the acceptable bioequivalence limits of 80 to 125%. Adhesion comparison showed the non-inferiority of the test formulation. Based on ANVISA’s regulatory requirements, the test and reference formulations were considered bioequivalent and could be interchangeable in clinical practice.

## 1. Introduction

Pain management has always been a critical aspect of healthcare, as millions of people worldwide live with chronic pain conditions that significantly impact their quality of life. In the search for effective and patient-friendly solutions, the development of transdermal delivery systems has emerged as a promising dosage form. Transdermal delivery systems offer some advantages for opioid administration, such as avoiding blood level peaks, allowing steady and continuous drug delivery, and reducing inconvenient side effects (such as nausea, vomiting, sedation, and respiratory depression). Another significant benefit of transdermal delivery systems is improved patient compliance, as they reduce the frequency of daily administrations (e.g., weekly patch administration) [1,2,3].

Buprenorphine is a synthetic lipophilic opioid and acts primarily as a partial agonist at mu-opioid receptors. This drug is indicated for the management of severe and persistent pain, requiring extended treatment with a daily opioid analgesic, and for which alternative treatment options are inadequate. Buprenorphine provides 30 to 50 times more analgesia than morphine and, due to its strong analgesic properties, it has proven effective in reducing chronic and neuropathic pain in various medical conditions (e.g., cancer patients or patients with severe arthropathies) [4,5,6].

Problems with buprenorphine oral formulations, such as poor gastrointestinal absorption and low systemic bioavailability due to both extensive first-pass gut wall and hepatic metabolism, have led to inadequate pain relief [7]. Due to its strong analgesic potential, new alternatives for the administration of buprenorphine have been developed by pharmaceutical companies, including sublingual or injectable formulations, subdermal implants, mucoadhesive buccal films, and transdermal patches [2]. Among the advantages of using buprenorphine transdermal patches is the convenient dosing interval, which allows once-weekly patch application, in contrast with oral opioids that may require multiple daily administrations [8]. Additionally, buprenorphine transdermal systems avoid extensive first-pass metabolism, resulting in improved bioavailability and reduced potential for drug–drug interactions [9].

Buprenorphine transdermal patches provide drug release for 7 days, with a steady state being achieved during the first application, by the third day of use. In Brazil, three strengths (5, 10, and 20 µg/h) are registered for buprenorphine transdermal patches (marketed as Restiva^®^) providing dose proportionality in terms of the total buprenorphine exposure following a 7-day application regimen. Transdermal delivery studies have demonstrated that intact human skin is permeable to buprenorphine, and its metabolism in the skin is not significant. The bioavailability of the buprenorphine patch is approximately 15% when compared to intravenous administration. A study conducted in healthy subjects showed that the pharmacokinetics of buprenorphine patches are similar when applied to the upper outer arm, upper chest, upper back, or the side of the chest. Considering both sides of the body, these four sites provide eight possible application sites, promoting rotation and reducing the potential for skin reactions in multiple patch application regimens [10,11,12].

Given the importance of generic versions for the transdermal drug delivery systems of opioid drugs, this study assessed the bioequivalence, skin adhesion performance, and tolerability of two buprenorphine patch formulations to meet Brazilian regulatory requirements for a generic product registration [13]. The comparison between the test and reference transdermal patch formulations involved evaluating pharmacokinetic profiles, bioequivalence, and skin adhesion performance to ensure that both products exhibit similar rate and extent of absorption, as well as non-inferiority in terms of adhesion within the dosing interval.

## 2. Materials and Methods

### 2.1. Ethical Aspects and Good Clinical Practices

All the phases of the bioequivalence and adhesion study were conducted at the Instituto de Ciências Farmacêuticas de Estudos e Pesquisas (Goiânia, Goiás, Brazil), a bioequivalence center certified by ANVISA. The protocol was evaluated and approved by the Research Ethics Committee at the Instituto de Ciências Farmacêuticas de Estudos e Pesquisas (Goiânia, Goiás, Brazil), with protocol number 4,550,668 (approved on 22 February 2021). Informed consent was obtained from all the subjects before the commencement of the study procedures. The study adhered to the principles of Good Clinical Practices [14], ethical standards for research involving human subjects as outlined in the Declaration of Helsinki [15], the current ethical regulations in Brazil (Resolution Number 466/2012, Ministry of Health, Brasília, Brazil) [16], and ANVISA requirements for bioequivalence studies [13].

### 2.2. Study Design and Sample Size

The study was designed as a single-center, randomized, balanced, single-dose, 2-period, 2-treatment, and 2-sequence crossover design. The participants were given either the test or the reference formulations separated by a 29-day washout period. Given the limited information published regarding the in vivo variability of buprenorphine transdermal patches, a 2-stage adaptive design was proposed. Therefore, if a sufficiently reliable power (≥80%) was achieved in the first stage, the study would be concluded to avoid the unnecessary exposure of additional subjects. Otherwise, the information obtained in the first stage would be used to accurately estimate the required sample size for the second stage. Method B described by Potvin et al. (2008) [17] would be considered with an adjustment of the significance level for the statistical analysis.

For the first stage, the sample size was estimated based on the within-subject variability (CV_ws_) obtained from a pilot study (internal data), which was found at approximately 27%. Additionally, an expected treatment difference of 7.5%; a significance level (α) of 2.94%, adjusted considering the two-stage approach; and a desired statistical power of 80% were considered. To ensure the robustness of the statistical results, potential dropouts, and non-compliance, additional subjects were also factored into the sample size. A sample size of 76 subjects was considered adequate for the bioequivalence analysis and skin adhesion performance.

### 2.3. Study Population

Seventy-six healthy subjects (38 males and 38 non-pregnant, non-lactating females) were selected with ages ranging from 18 to 50 years, and a body mass index (BMI) between 18.50 and 28.63 kg/m^2^. Regarding the inclusion and exclusion criteria, the subjects had not previously participated in another clinical trial nor donated blood during the preceding six months and had no history of alcohol or drug abuse. In addition, all the subjects had good health conditions or the absence of significant diseases after assessing their medical history, vital signs, physical examinations, electrocardiogram (ECG), and routine laboratory tests. Moreover, the subjects’ clinical histories and episodes of gastrointestinal disorders were assessed, as well as any medications that could potentially interfere with buprenorphine pharmacokinetics. Due to the COVID-19 pandemic, ongoing at the time of the study conduction, an additional requirement was a negative result for the RT-qPCR test for SARS-CoV-2. Additionally, subjects with skin markings, such as pigmentation issues, scars, excessive hairiness, or tattoos in the patch application area, were excluded since they may interfere with patch adhesion performance and, consequently, drug absorption.

To guarantee protocol compliance, the subjects remained hospitalized for approximately 184 hours at the clinical facility to attend all the procedures related to blood sample collections, adhesion assessment, and safety monitoring.

### 2.4. Patch Formulations

The test formulation was a buprenorphine transdermal patch of 20 µg/h strength (batch No. 40565/40566, expiry date: April 2022), manufactured by Amarin Technologies S.A. (Buenos Aires, Argentina) and imported by Adium S.A. (São Paulo, Brazil). The reference formulation was Restiva^®^ (marketed in other countries as BuTrans^®^ or Norspan^®^), a buprenorphine transdermal patch of 20 µg/h strength (batch No. 70440C101, expiry date: March 2022) manufactured by Lohmann Therapie Systeme A.G. (Andernach, Germany) and imported by Mundipharma (São Paulo, Brazil). Before the in vivo study, in vitro assays were conducted with the same batches to guarantee the pharmaceutical equivalence and release profile similarity of both products.

Transdermal patches need to offer sufficient driving force to allow the drug to diffuse through the skin. In consequence, the drug loaded in the device (drug content, expressed as mg per patch) exceeds the drug delivered (dose, expressed as µg/h). A residual of the drug remains in the patch after use. In Table 1, the attributes of both test and reference formulations were compared.

**Figure 1:**
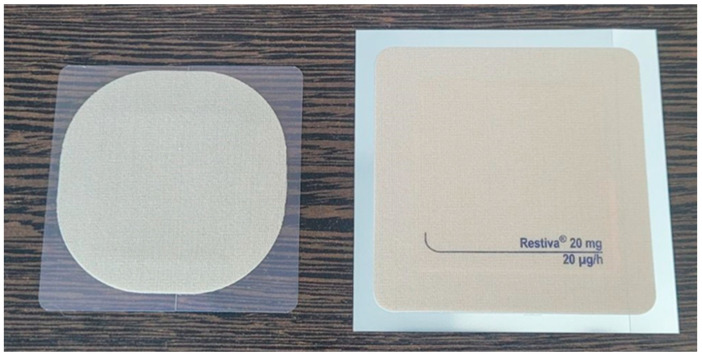
Photograph of the test (**left**) and reference (**right**) formulations used in the study.

Since the API is an opioid substance, this is crucial because less drug remain in the used test patch, reducing the potential for abuse. As shown in Figure 1, the smaller size of the test patch offers an advantage in terms of adhesion performance compared to Restiva^®^ A larger TDS may be more subjected to conformational or torsional strains/flexion due to anatomical curvatures, increasing the risk of early patch dislodgement.

It is important to reinforce that the test formulation has a lower drug content in a smaller patch size compared to the reference product Restiva^®^ for the same strength (in this study, 20 µg/h), being in line with FDA guidance on residual drug content for transdermal products [18]. Therefore, the test formulation is pharmaceutically more efficient than Restiva^®^, considering the products promote the same dose release (20 µg/h). 

As recommended in the FDA product-specific guidance for buprenorphine transdermal patch [19], the need for in vivo testing for the 5 µg/h and 10 µg/h strengths was waived due to the proportional similarity of the formulations (excipients and dimensions), identical release mechanism and manufacturing site, and comparable in vitro release profiles with the bio-batch (20 µg/h) strength.

### 2.5. Administration and Diet

The prescribing information for the reference product [10] recommends the administration of a buprenorphine transdermal patch to non-irritated and intact skin on the upper outer arm, upper chest, upper back, or the side of the chest (mid-axillary line, 5th intercostal space). To standardize the patch application, the upper back region was chosen for the administration in the periods and both the left and right sides were considered for each formulation application following the subject randomization list.

Before administration, the subject’s skin was checked to ensure it was clean, dry, and hairless, without any powder, oil, moisturizer, or lotion that may interfere with the adhesion performance. Moreover, the skin was examined for cuts, rashes, irritations, inflammations, scars, tattoos, vitiligo, or other local abnormalities that could interfere with the adhesion performance assessment.

Each study period began with a minimum overnight fasting period of eight hours. After patch application, the subjects refrained from consuming any food for four hours and were advised to limit their water intake within two hours before and following drug administration. To ensure consistency across the treatment groups, the dietary regimen, encompassing both food and beverages, remained standardized for all the participants throughout both periods of the study. Furthermore, the consumption of alcoholic beverages, as well as food or drinks containing caffeine or xanthine (such as coffee, tea, chocolate, cola, or guarana-based soft drinks), was prohibited in the 48 h before participation in the study.

During the study, the subjects were hospitalized for approximately 184 hours in both study periods. To ensure accuracy in adhesion performance measurement, procedures were implemented to avoid patch displacement during the dosing interval. These procedures included standardizing shower times by prohibiting the first shower 3 hours before and 12 hours after patch application. Additionally, the water temperature was controlled between 19 and 29 °C, and the duration of each shower was limited to 10 min. It was also prohibited to wash the skin region where the patch was applied and only a trained nurse was allowed to dry the area. These measures helped to reduce the risk of bias in the adhesion performance assessment.

### 2.6. Rescue Medication (Opioid Antagonist)

Based on the FDA product-specific guidance for buprenorphine transdermal patches [19], an opioid antagonist should be administered to minimize opioid-related adverse events in bioequivalence studies. The opioid antagonist should be administered before dosing to achieve the adequate blockade of opioid receptors. In this way, naltrexone hydrochloride 50 mg tablet was chosen as the opioid antagonist, administered at four different moments: one hour before dosing, and 12, 72, and 144 h after patch application in both study periods. If necessary (e.g., in the case of an episode of opioid intoxication), the medical team could adopt additional doses of naltrexone or naloxone 0.4 mg/mL intravenous to guarantee the safety of the subjects.

### 2.7. Sampling and Bioanalysis

For bioanalysis, a total of 25 blood samples were collected at 0 (before drug administration), 3, 6, 12, 18, 24, 36, 48, 54, 60, 66, 72, 78, 84, 96, 108, 120, 132, 144, 156, 168, 192, 216, 240, and 288 h after patch application. Blood samples were collected in tubes containing heparin (anticoagulant) and centrifuged at 3000 rpm for 5 min at 4 °C. Then, the resulting plasma (approximately 1.8 mL) was carefully separated, transferred to cryogenic tubes, and labeled. Finally, these samples were stored at −20 °C until bioanalysis.

The plasma samples were analyzed using validated ultra-high-performance liquid chromatography-tandem mass spectrometry (UHPLC-MS/MS) to obtain buprenorphine concentrations for each subject and period. The system includes an Agilent 1200 Series (Agilent Technologies Inc., Santa Clara, CA, USA) and an API 6500 MS/MS (Sciex, Framingham, MA, USA). The analytes were extracted from the plasma using a liquid–liquid method and buprenorphine-d4 (USP, Rockville, MD, USA) as an internal standard (IS). To avoid inter-assay variations, all the samples from the same participant were assessed in the same analytical run.

An amount of 30 µL of each sample was injected onto a Zorbax Eclipse XDB-Phenyl (4.6 × 150 mm; 3.5 µm) (Agilent Technologies Inc., Santa Clara, CA, USA) column, maintained at 30 °C. The mobile phase consisted of a mixture (75:25) of (A) methanol and (B) ammonium acetate 2 mM solution (*v*/*v*, with 0.025% formic acid). The flow rate was 0.7 mL/min in an isocratic performance. The detection of buprenorphine was carried out in the mass spectrometer with the positive electrospray ionization multiple-reaction monitoring mode set to transmit at *m*/*z* 468.142 → 396.200 for buprenorphine and *m*/*z* 472.169 → 400.300 for buprenorphine-d4 (IS).

The analyte concentrations were calculated through interpolation on the calibration curve, and the linearity range used was from 10 to 1000 pg/mL. The bioanalytical method was validated in compliance with ANVISA guidance for bioanalytical method validation [20], including the evaluation of selectivity, concomitant medication interference, matrix effect, carry-over, calibration curve, precision, accuracy, reinjection reproducibility, and the stabilities of buprenorphine under different conditions.

### 2.8. Pharmacokinetic and Statistical Analysis

The pharmacokinetic parameters were obtained from the curves of buprenorphine plasma concentration versus time and statistically compared for bioequivalence using Phoenix WinNonlin™ version 6.4 (Princeton, NJ, USA). The calculation of the area under the curve from zero to the last quantifiable concentration (AUC_0–t_) was performed using the trapezoidal method, and the area under the curve from zero to infinity (AUC_0–inf_) was calculated by the formula AUC_0–t_ + (Cn/kel), where Cn was the last quantifiable plasma concentration. The elimination constant (kel) was determined by analyzing the elimination phase of the graph depicting the log plasma concentration versus time. The elimination half-life (t_1/2_) was defined using the equation t_1/2_ = Ln (2)/kel and the maximum plasma drug concentration (Cmax) was obtained directly from the experimental data, as well as the time of the occurrence of Cmax (tmax).

Based on ANVISA requirements for bioequivalence studies involving transdermal patches [13], the following two partial AUC (pAUC, a representative metric of the shape of the curve) were necessary as primary endpoints in addition to the conventional metrics (Cmax and AUC_0–t_):

- Early pAUC: the area under the curve from zero to half of the dosing interval;
- Terminal pAUC: the area under the curve from the half of the dosing interval to the last quantifiable plasma concentration.

Thus, for this study, the early pAUC was calculated from 0 to 84 h, and the terminal pAUC was estimated from 84 to 288 h.

To evaluate bioequivalence, predefined acceptance criteria (set in the range of 80.00 to 125.00%) were applied to the 90% confidence interval for the ratio of test and reference formulations (T/R) for the log-transformed data of Cmax and AUC (AUC_0–t_ and pAUC). An analysis of variance (ANOVA) test was conducted to evaluate the effects of sequence, period, and treatment (as fixed effects) on these parameters.

### 2.9. Adhesion Performance Assessment

Skin adhesion was measured at 0 (immediately after the patch application), 24, 48, 72, 96, 120, 144, and 168 h after the application of the patch formulations. Skin adhesion percentages were measured using standardized molds made from a transparent plastic material, which featured small squares for counting detached areas. Two different models were used for the test and reference formulations due to their varying shapes and dimensions (30.96 cm^2^ versus 51.58 cm^2^) (Figure 1). Adhesion assessment involved recording the number of marked areas on the transparent mold at each evaluation point. The total area of all the marked squares was used to calculate the detached area.

The percentage of the adhered area was estimated based on the difference between the total area and the detached area. The percentage of the adhesion measured at the end of the dosing interval (168 h) was considered the primary endpoint for skin adhesion comparison. Based on the EMA guideline [21], the following criteria were considered for adhesion comparison:

- If the reference formulation showed high skin adhesion (≥90%): the test formulation would be considered equivalent if the lower limit of the 90% confidence interval (CI) of the mean percentage of adhesion for the test, at the end of the dosing interval (168 h), is greater than 90%;
- If the reference formulation showed low skin adhesion (<90%): the test formulation would be considered non-inferior if the lower limit of the 90% CI for the difference in adhesion (test–reference), using the percentage of adhesion as a continuous variable, is greater than the margin of non-inferiority (–δ). For statistical analysis, the non-inferiority margin was set as minus 10% (−10%).

To maintain a record for future consultations by regulatory agencies or sponsors, an informative photographic record was made for each adhesion assessment, and all the images were adequately stored in the study files.

### 2.10. Safety Measures

All the participants were continuously and carefully monitored during all the phases of the study. Safety was assessed by monitoring the baseline and ongoing vital signs including temperature, blood pressure, heart rate, and respiratory rate throughout the study. Additionally, laboratory tests (such as hematology, urinalysis, and blood biochemistry), physical examinations, and electrocardiograms (ECGs) were conducted at the beginning and conclusion of the study. Adverse events were assessed by the nursing and medical staff throughout the entire study. The subjects were instructed regarding the need to immediately report any undesirable symptoms or medical conditions during the study or after the hospitalization period. Adverse events were graded as mild, moderate, or severe, and their causality to the drug was determined by the medical staff as suspected or not suspected.

## 3. Results

### 3.1. Demographic Data

After the medical history assessment, verification of vital signs, physical examination, electrocardiogram, and routine laboratory tests, all the subjects showed good health conditions and the absence of significant diseases. Seventy-six (76) healthy subjects were enrolled in the study and fifty-two subjects (24 women and 28 men) completed the two periods, being included in the pharmacokinetic, adhesion, and statistical analysis. Table 2 shows the demographic characteristics of the subjects who completed all the phases of the study.

Before drug administration in the first period, one subject was withdrawn due to a positive result for the β-HCG test, one subject due to altered ECG, one subject due to skin injuries (acnes) in the dorsal region, two subjects due to the use of prohibited drugs (benzodiazepine and secnidazole), and four subjects due to abnormal prostate-specific antigen levels. During the first period, one subject was withdrawn due to inadequate social behavior, one subject due to vomit episodes, two subjects due to skin injuries (pruritus scapular), one subject due to oropharyngeal edema, one subject due to lower abdominal pain (including dysuria), two subjects due to hypertension crisis, and two subjects abandoned the study due to personal reasons. In the second period, one subject was withdrawn due to a hypertension crisis, one subject reported pain in the lower abdomen (including dysuria), and three subjects did not attend the hospitalization.

### 3.2. Pharmacokinetic Analysis

**Figure 2:**
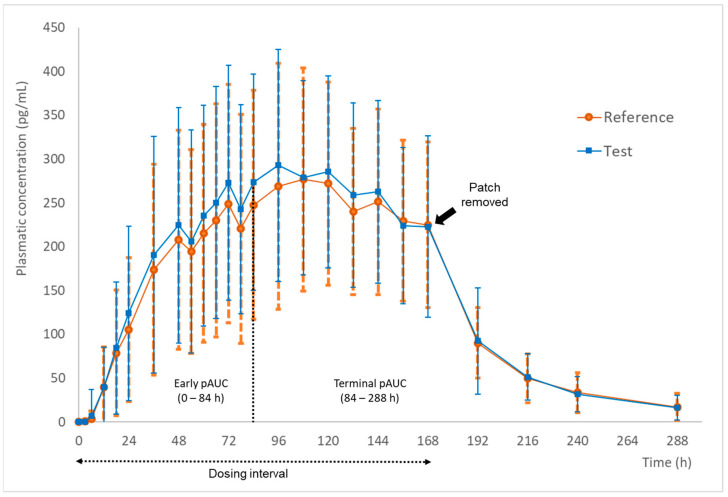
Mean plasma concentration vs. time (± SD) curves after the administration of the test (buprenorphine transdermal patch 20 µg/h) and the reference (Restiva^®^ 20 µg/h) formulations in healthy male and non-pregnant female subjects (*n* = 52). The patches were removed 168 h (7 days) after the application, and the blood samples were collected up to 288 h.

Figure 2 shows the mean plasma concentration versus time calculated for the 52 subjects who successfully completed both study periods and were included in the pharmacokinetic analysis. It is possible to note that the sampling scheme was suitable to elucidate the absorption phase within the dosing interval (0–168 h), as well as the drug elimination (168–288 h) after the patch removal. For pAUC metrics, the early and terminal pAUC were estimated from 0 to 84 h (half of the dosing interval) and 84 to 288 h, respectively.

The pharmacokinetic metrics of buprenorphine for both formulations are described in Table 3. As previously mentioned, the pAUC metrics (AUC_0–84_ and AUC_84–t_) were also calculated to attend to the ANVISA requirements for bioequivalence studies with transdermal patches, ensuring the products are equivalents in the different moments of the dosing interval.

### 3.3. Bioequivalence Assessment

Table 4 describes the test/reference geometric mean ratios obtained for the pharmacokinetic metrics Cmax, AUC_0–t_, AUC_0–inf_, AUC_0–84_, and AUC_84–t_ along with the corresponding 90% CIs, CV_ws_, and statistical power of the bioequivalence analysis. Since the study achieved sufficient power (≥80%) with 52 subjects in the first stage, additional subjects were not exposed unnecessarily.

Considering that the 90% CIs of the test/reference geometric mean ratios of all the pharmacokinetic metrics were within the bioequivalence acceptance range of 80.00–125.00% established by ANVISA [13], both patch formulations (test and reference) were considered bioequivalent in terms of the rate and extent of absorption.

### 3.4. Skin Adhesion Performance

**Figure 3:**
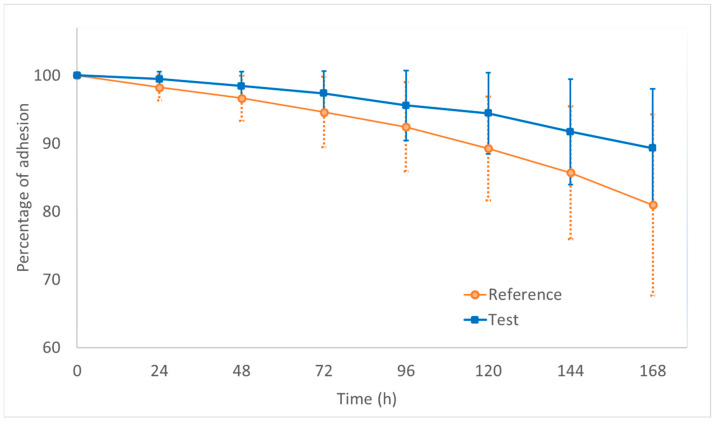
Adhesion assessment within the dosing interval (7 days) for the test (squares) and reference (empty circles) patch formulations. Data are expressed as mean ± SD (*n* = 52).

Figure 3 shows the mean adhesion percentage for each patch formulation over the dosing interval (7 days or 168 h) for all the subjects who concluded the study. It is possible to observe the adhesion profile of the patch formulations at different moments after application on the subjects’ skin.

Since the reference product showed a percentage of adhesion lower than 90% at the end of the dosing interval (7th day), a non-inferiority test was performed to statistically compare both the test and reference patch formulations. Table 5 shows the statistical results, non-inferiority margin, and obtained power.

Non-inferiority was demonstrated for the test formulation since the mean difference between the formulations was estimated at 8.37% and the lower limit of the CI for the difference was estimated at 4.71%, thus, greater than the non-inferiority margin (−10%).

### 3.5. Adverse Events

From the 76 subjects enrolled in the study, 123 adverse events were reported and all of them were classified as mild regarding intensity and likely or unlikely regarding causality. Table 6 describes the reported events with an incidence ≥2%.

No serious adverse events occurred during the study, and there was one report of pregnancy before drug administration in the first period. This subject was immediately withdrawn from the study.

## 4. Discussion

### 4.1. Pharmacokinetics and Bioequivalence Assessment

The present study compared the bioavailability between two formulations (test and reference) of buprenorphine transdermal patches. It was possible to elucidate the pharmacokinetic profiles of buprenorphine within the dosing interval, as both the transdermal patches delivered the drug consistently over the seven-day application period. Moreover, the study design was adequate to demonstrate the elimination of the drug when the products were removed from the skin.

Regarding the pharmacokinetic metrics, some results were consistent with the study BP96-0304 reported in the Clinical Pharmacology and Biopharmaceutics Review (issued by FDA) [12] for the product BuTrans^®^. In this document, a mean of Cmax of 307 (±99) pg/mL for 20 µg/h strength administration was reported, and in our study, we found Cmax of 351 (±135) pg/mL and 330 (±142) pg/mL for the test and reference patch formulations, respectively. It is possible to observe a variation when comparing the tmax values, once the FDA document reports 71 (±9) h for BuTrans^®^ 20 µg/mL and we found 112 (±37) h and 111 (±33) h for the test and the reference formulations, respectively. This discrepancy could be attributed to the differences in the sampling methods employed in each study, especially in the first days of the buprenorphine pharmacokinetic curve. In the study BP96-0304, the patch was removed 72 h after the application, while in our study, we adopted a protocol to keep the products attached to the skin during the entire 168-hour dosing interval. Regarding the elimination half-life (t_1/2_), in our study, this parameter was 38 (±15) h for the test formulation and 41 (±16) h for the reference product, while the study with BuTrans^®^ reports 24 (±7) h. This variability could also have arisen from the differences in the sampling methodologies between the compared studies, herein mainly related to the blood samples collected in the elimination phase of the pharmacokinetic profile. In our study, the blood samples were collected up to 288 h post-dose and, in the study BP96-0304, only up to 144 h. Also, differences in the sensitivity (lower limit of quantification (LLOQ)) of the bioanalytical method could have influenced the estimation of the t_1/2_. Our study established an LLOQ of 10 pg/mL for the quantification of the plasma samples, while an LLOQ of 25 pg/mL was reported for the study BP96-0304, denoting its lower sensitivity.

When our results are compared with the study BP97-0501 (also reported in the same FDA Clinical Pharmacology and Biopharmaceutics Review) [12], tmax and t_1/2_ are more consistent once the sampling and the duration of patch attachment to the skin were more similar. It was reported to be 90 (±13) h and 35 (±4) h for tmax and t_1/2_, respectively, for 20 µg/h patch administration. These values are similar to those described above in Table 3. On the other hand, the Cmax (471 pg/mL) reported for the study BP97-0501 is very different than the one observed in the present study because the studies have a relevant difference in terms of washout. For the study BP97-0501, a washout of 2 to 10 days was reported, while we have set up at least 29 days as a washout period in our study, aiming to avoid residual effects between the treatments.

These comparisons are crucial to demonstrate that the present bioequivalence study was correctly designed and suitable to elucidate the pharmacokinetics of buprenorphine when administered as a transdermal patch formulation.

Regarding the bioequivalence assessment, the 90% CIs for the ratio of the geometric means (test/reference) of logarithmically transformed pharmacokinetic parameters were comprised within the ranges of 101.50 to 111.80% for Cmax, 100.54 to 110.88% for AUC_0–t_, 103.21 to 118.57% for AUC_0–84_, and 97.39% to 108.28% for AUC_84–t_. All these values were within the predefined range of 80.00% to 125.00%, demonstrating sufficient statistical power to detect differences between the treatment groups. To the best of our knowledge, this is the first study considering partial AUCs as primary endpoints for bioequivalence assessment between two formulations of buprenorphine transdermal patches.

### 4.2. Adhesion Performance

The skin adhesion performance of the test and reference formulations was compared at the end of the dosing interval (7 days after the patch application). Visually, the test formulation demonstrated better adhesion compared to the reference product throughout the dosing interval (Figure 3).

For the statistical comparison, the criteria for evaluating the adhesion of the reference formulation were based on the EMA guideline [21]. Since the reference formulation exhibited low skin adhesion (<90%) at the end of the dosing interval, a non-inferiority test was applied to compare the formulations. As shown in Table 5, the mean adhesion for the reference product was 81%, while for the test formulation, it was 89%. The mean difference between the formulations was calculated at 8.37%, with the lower limit of the CI estimated at 4.71%, which is above the non-inferiority margin (−10%). Furthermore, since the lower limit also exceeds zero, it can be concluded that the skin adhesion of the test formulation was indeed superior to that of the reference product. Finally, the power of the statistical test was estimated at nearly 100%, further supporting the robustness of the results.

It is relevant to note that, for adhesion assessment, both formulations (test and reference) were administered to the same subject at different periods (using contralateral sides) to reduce the influence of inter-subject variability related to skin characteristics.

### 4.3. Safety Outcome

A total of 123 adverse events were reported for both the test and reference formulations by the 76 subjects enrolled in the study. The most common were headache (31%), leukocyturia (11%), and nausea (9%). Two of them (headache and nausea) are adverse events reported on the product label [10] as very common during the use of buprenorphine transdermal patches.

Fortunately, no serious adverse events occurred during the study. There was one report of pregnancy before the drug administration in the first period and the woman was immediately withdrawn from the study and medical assistance for prenatal was provided.

It is important to mention that based on the FDA product-specific guidance for buprenorphine transdermal patch [19], in addition to the bioequivalence and adhesion studies, it is necessary to perform a skin irritation/sensitization study to guarantee the safety of the generic formulation. The test formulation was compared to the reference product in another clinical study and proven to be safe also in terms of skin irritation/sensitization.

## 5. Conclusions

This study adequately characterized the pharmacokinetic profile and adhesion performance of both the test and reference patch formulations, compliant with the ANVISA regulatory requirements. Given the similarity in the bioavailability and non-inferiority of skin adhesion, the test formulation was considered bioequivalent to the reference product (Restiva^®^). Therefore, both patch formulations are expected to produce the same therapeutic response, making them interchangeable in clinical practice.

## Figures and Tables

**Table 1:** Characteristics of the patch formulations.

Characteristic	Test	Reference
Drug content (mg per patch)	9.75	20
Dose delivered (µg/h)	20	20
Dose delivered after 7 days (mg per patch)	3.36	3.36
Patch delivery efficiency (%)	34.5	16.8
Active patch area (cm^2^)	15.6	25
Total patch area (cm^2^)	30.96	51.58

**Table 2:** Demographic characteristics of the study subjects.

Characteristic	Descriptive Statistics *n* = 52
Age (years)	
Mean (±SD)	33.9 (±9.07)
Range	19–50
Weight (kg)	
Mean (±SD)	68.1 (±8.33)
Range	53.4–91.4
Height (m)	
Mean (±SD)	1.67 (±0.08)
Range	1.49–1.89
BMI (kg/m^2^)	
Mean (±SD)	24.4 (±2.66)
Range	19.1–28.6
Gender (*n* [%])	
Male	28 (54%)
Female	24 (46%)

**Table 3:** Pharmacokinetic metrics of buprenorphine for the test (buprenorphine transdermal patch 20 µg/h) and reference (Restiva^®^ 20 µg/h) formulations administered in healthy subjects (*n* = 52). Data expressed as mean (±SD).

Parameter	Test	Reference
Cmax (pg/mL)	351.77 (±135.22)	330.19 (±142.87)
AUC_0–t_ (pg/mL·h)	43,980.27 (±17,920.81)	41,893.76 (±18,437.88)
AUC_0–84_ (pg/mL·h)	14,268.85 (±8270.47)	13,033.92 (±7855.32)
AUC_84–t_ (pg/mL·h)	29,821.55 (±11,366.57)	28,974.53 (±11,586.25)
AUC_0–inf_ (pg/mL·h)	45,685.47 (±18,183.43)	43,312.66 (±19,202.20)
tmax (h)	112.39 (±36.64)	111.81 (±32.61)
t_1/2_ (h)	38.08 (±14.62)	40.60 (±15.70)

Cmax, maximum plasma concentration; AUC_0–t_, area under the concentration–time curve from zero to 288 h; AUC_0–inf_, area under the concentration–time curve extrapolated to infinity; AUC_0–84_, area under the concentration–time curve from zero to 84 h; AUC_84–t_, area under the concentration–time curve from 84 to 288 h; tmax, time to Cmax; t_1/2_, elimination half-life.

**Table 4:** Geometric mean ratio, confidence intervals (90%), CV_ws_, and power (*n* = 52).

Parameter *	GMR (%)	90% CI	CV_ws_ (%)	Power (%)
Cmax	106.53	101.50–111.80	14.59	99.98
AUC_0–t_	105.58	100.54–110.88	14.80	99.99
AUC_0–84_	110.62	103.21–118.57	21.08	84.37
AUC_84–t_	102.69	97.39–108.28	16.03	100.0
AUC_0–inf_	104.46	99.45–109.73	14.68	100.0

* parameters logarithmically transformed; GMR, geometric mean ratio; CI, confidence interval; CV_ws_, coefficient of variation (within subject).

**Table 5:** Non-inferiority test for the mean percentage of adhesion at the end of the dosing interval (7 days after patch application) comparing the test and the reference formulations (*n* = 52).

Treatment/Statistics	Mean	SE	SD	90% CI
Reference	80.95	1.84	13.30	77.25–84.65
Test	89.32	1.20	8.67	86.90–91.73
Difference (Test-Reference)	8.37	2.20	-	4.71–12.02
Non-inferiority margin (–δ)	−10%			
Statistical power	100%			

SE, standard error; SD, standard deviation; CI, confidence interval.

**Table 6:** Incidence of adverse events (*n* = 76 subjects; 123 adverse events reported).

Adverse Event	Incidence *n* (%)	Causality	Intensity
Headache	38 (31%)	Likely	Mild
Leukocyturia	13 (11%)	Unlikely	Mild
Nausea	11 (9%)	Likely	Mild
Dizziness	7 (6%)	Likely	Mild
Hematuria	7 (6%)	Unlikely	Mild
Colic	6 (5%)	Unlikely	Mild
Vomit	6 (5%)	Likely	Mild
Hypertension	4 (3%)	Likely	Mild
Backache	3 (2%)	Likely	Mild
Eosinophilia	3 (2%)	Unlikely	Mild
Others	<2%	-	-
